# Cancer Genes Hypermethylated in Human Embryonic Stem Cells

**DOI:** 10.1371/journal.pone.0003294

**Published:** 2008-09-29

**Authors:** Vincenzo Calvanese, Angelica Horrillo, Abdelkrim Hmadcha, Beatriz Suarez-Álvarez, Agustín F. Fernandez, Ester Lara, Sara Casado, Pablo Menendez, Clara Bueno, Javier Garcia-Castro, Ruth Rubio, Pablo Lapunzina, Miguel Alaminos, Lodovica Borghese, Stefanie Terstegge, Neil J. Harrison, Harry D. Moore, Oliver Brüstle, Carlos Lopez-Larrea, Peter W. Andrews, Bernat Soria, Manel Esteller, Mario F. Fraga

**Affiliations:** 1 Cancer Epigenetics Group, Spanish National Cancer Research Centre (CNIO), Madrid, Spain; 2 Andalusian Center for Molecular Biology and Regenerative Medicine (CABIMER), Seville, Spain; 3 Unidad de Histocompatibilidad, HUCA, Oviedo, Spain; 4 Cancer Epigenetics and Biology Program (PEBC), Catalan Institute of Oncology (ICO), Barcelona, Spain; 5 Andalusian Stem Cell Bank (BACM)/University of Granada, Instituto de Investigaciones Biomédicas, Parque Tecnológico de la Salud, Granada, Spain; 6 S. de Genética Médica y Molecular, Hospital Universitario La Paz, Madrid y CIBERER, Centro de Investigación Biomédica en Red de Enfermedades Raras, Madrid, Spain; 7 Department of Histology, University of Granada, Granada, Spain; 8 Institute of Reconstructive Neurobiology, LIFE & BRAIN Center, University of Bonn and Hertie Foundation, Bonn, Germany; 9 Centre for Stem Cell Biology and the Department of Biomedical Science, University of Sheffield, Sheffield, United Kingdom; 10 Department of Immunology and Oncology, Centro Nacional de Biotecnología/CSIC, Darwin 3, Cantoblanco, Madrid, Spain; Netherlands Cancer Institute, Netherlands

## Abstract

Developmental genes are silenced in embryonic stem cells by a bivalent histone-based chromatin mark. It has been proposed that this mark also confers a predisposition to aberrant DNA promoter hypermethylation of tumor suppressor genes (TSGs) in cancer. We report here that silencing of a significant proportion of these TSGs in human embryonic and adult stem cells is associated with promoter DNA hypermethylation. Our results indicate a role for DNA methylation in the control of gene expression in human stem cells and suggest that, for genes repressed by promoter hypermethylation in stem cells *in vivo*, the aberrant process in cancer could be understood as a defect in establishing an unmethylated promoter during differentiation, rather than as an anomalous process of *de novo* hypermethylation.

## Introduction

In the course of embryonic development, cells are initially totipotent but, after a few divisions, begin to lose potency and are transformed into pluripotent cells, finally becoming terminally differentiated somatic cells. The progressive loss of potency during differentiation has fundamental implications for disease because recovery of pluripotency through nuclear reprogramming is one of the major challenges in regenerative medicine [1], and because disruption of the developmental process that gives rise to a terminally differentiated somatic cell from its corresponding progenitor cell may result in malignant transformation [2].

Differentiation of human embryonic stem cells (hESCs) requires the repression of transcription factors involved in maintaining pluripotency and the activation of developmental genes. Both processes are directed by specific epigenetic mechanisms. An example of the first type is the promoter hypermethylation-dependent repression of pluripotency-maintaining genes such as NANOG and OCT4 as stem cells differentiate [3]. So far, activation of developmental genes during stem cell differentiation upon DNA methylation has been less thoroughly studied. Instead, these developmental genes have been reported as being in a repressed state during early stages of development due to the establishment of a specific pattern of histone modification, termed “bivalent domains”, which consists of large regions of H3 lysine 27 methylation harboring smaller regions of H3 lysine 4 methylation [4]. This chromatin-repressive status is mediated by the Polycomb group of proteins [5], [6] and is thought to predispose to aberrant promoter hypermethylation in cancer [7]–[9]. The finding that treatment of hESCs with the demethylating drug 5′-Aza-2′-deoxycytidine causes cardiac differentiation and gene reactivation [10] prompted us to consider whether promoter DNA methylation could contribute to the establishment and maintenance of specific-gene repression in hESCs. To establish its existence and its putative relationship with aberrant promoter hypermethylation in cancer, we compared the promoter DNA methylation pattern of a panel of 800 cancer-related genes between hESCs and different types of terminally differentiated adult tissues and cancer cell lines.

## Results

### Promoter DNA methylation profiling in hESCs, normal differentiated tissues and cancer samples

**Figure 1:**
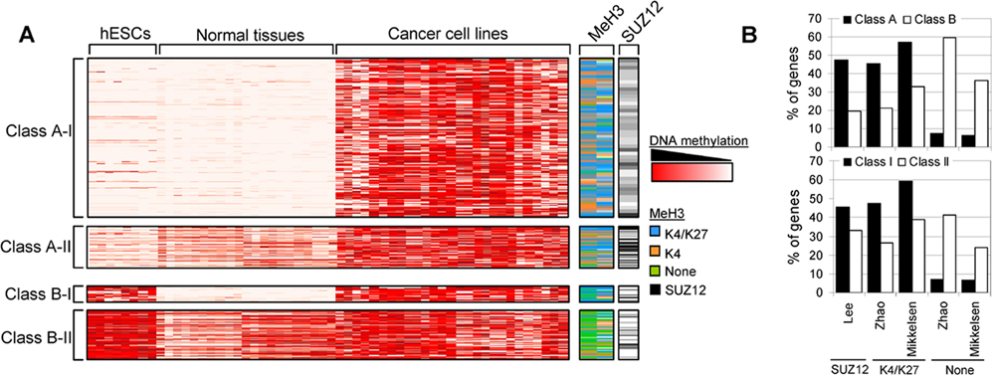
DNA methylation profiling in human embryonic stem cells (hESCs), normal primary tissues, and cancer cell lines. (A) Methylation profiles of Class A-I (350), A-II (94), B-I (20), and B-II (107) genes in hESCs (8), normal (21), and cancer (21) samples obtained by Illumina arrays. The methylation levels vary from fully methylated (red) to fully unmethylated (white). The right-hand columns show the methylation status of histone H3 and Polycomb occupancy of the same genes obtained from previously published data [5], [12], [16]. Blue, methylation at K4 and K27; orange, methylation at K4 alone; green, no methylation at K4 or K27; black, presence of the Polycomb protein SUZ12. (B) Enrichment of the Polycomb protein SUZ12, the bivalent chromatin signature (K4/K27) or the absence of both marks (none) in Class A and Class B genes (upper panel) and Class I and Class II genes (lower panel).

We used Illumina Goldengate Methylation Arrays© to compare the DNA methylation status of 1,505 sequences (from 807 genes) in eight independently isolated hESCs lines, 21 normal human primary tissues (NPTs) corresponding to six normal tissue types (NTTs) and 21 human cancer cell lines (CCLs) (see Methods). The genes included in the methylation arrays were chosen on the basis of their importance to cellular behavior and differentiation and included genes previously reported to be differentially methylated , as well as tumor suppressor genes, oncogenes and genes coding for factors involved in cell cycle check point [11]. We first selected the autosomal genes (766) from the arrays in order to exclude DNA methylation-dependent X chromosome inactivated genes. As previously reported, unsupervised clustering of the samples exclusively using the methylation signals of the autosomal genes (1,421 sequences) contained in the arrays enabled the correct classification of each sample within its corresponding group (hESC, NPT, or CCL) (**Figure 1A**, and see **Figure S1** in the online supplementary data for this article), thereby confirming that each group of samples has a specific DNA methylation signature [11]. We then attempted to classify the genes in the array in relation to their methylation status in the three types of sample analyzed (hESC, CCL, and NPT) (**Figure 1A**, and **Tables 1** and **S1**). We found that 65.31% (928/1,421) of the sequences were not frequently hypermethylated in hESCs (array signal≤0.7 in≥25% (2/8) of the samples) and that half of them (464/928) were frequently hypermethylated in CCLs (array signal>0.7 in≥25% (6/21) of the samples). The vast majority of these sequences (99.78%, 463/464) were unmethylated in at least one of the NTTs analyzed (array signal<0.3 in≥1/6 NTTs). This finding is consistent with the view that genes aberrantly hypermethylated in cancer (i.e., not hypermethylated in normal tissues) are not hypermethylated in hESCs [8]. We called this group of genes classical Class A cancer hypermethylated genes (**Tables 1** and **S1**).

**Table 1:** Classification of genes according to their promoter methylation status in hESCs, normal tissues, and CCLs, and proposed biological role for each group.

	Methylation in hESCs	Methylation in CCLs	Methylation in NTTs	Proposed biological role	Name of the category	Group of genes from Supplementary Table 1
1421 Sequences	Hypermethylated in hESCs>0.7 in≥2/8 samples	Hypermethylated in hCCLs>0.7 in≥6/21 samples	159 Sequences (11.19%) hypermethylated in all NTTs (>0.7 in 6/6 samples)	Genes constitutively hypermethylated	-	G1
			20 Sequences (1.41%) unmethylated in all NTTs (<0.3 in 6/6 samples)	Genes that become demethylated early during hESC differentiation or that become aberrantly hypermethylated during *in vitro* culture hESCs. Their hypermethylation might provide advantages to the cancer cells.	Class B-I	G2
		393 Sequences (27.66%)	107 Sequences (7.53%) sometimes unmethylated (≤0.3 signal in ≥1/6 and ≤5/6 samples)	Genes which demethylation during hESC differentiation might be important for lineage specification. Their hypermethylation might provide advantages to the cancer cells.	Class B-II	G3
	493 Sequences (34.69%)	Not hypermethylated in hCCLs	16 Sequences (1.13%) hypermethylated in all NTTs (>0.7 in 6/6 samples)	Genes that become frequently demethylated in cancer	-	G4
		Not>0.7 in≥6/21 samples	11 Sequences (0.77%) unmethylated in all NTTs (<0.3 in 6/6 samples )	Genes that become demethylated early during hESC differentiation. Their hypermethylation might not provide advantages to the cancer cells.	-	G5
		100 Sequences (7.04%)	39 Sequences (5.07%) sometimes unmethylated (≤0.3 signal in ≥1/6 and ≤5/6 samples)	Genes which demethylation during hESC differentiation might be important for lineage specification. Their hypermethylation might not provide advantages to the cancer cells.	-	G6
	Not hypermethylated in hESCs not>0.7 in≥2/8 samples	Hypermethylated in hCCLs>0.7 in≥6/21 samples	1 Sequences (0.07%) hypermethylated in all NTTs (>0.7 in 6/6 samples)	Genes hypermethylated early during hESC differentiation. Their hypermethylation should not provide advantages to the cancer cells.	-	G7
			350 Sequences (24.63%) unmethylated in all NTTs (<0.3 in 6/6 samples)	Genes constitutively unmethylated during normal development. Their aberrant hypermethylation provide advantages to the cancer cells.	Class A-I	G8
		464 Sequences (32.65%)	94 Sequences (6.61%) sometimes unmethylated (≤0.3 signal in ≥1/6 and ≤5/6 samples)	Genes which hypermethylation during hESC differentiation might be important for lineage specification. Their aberrant hypermethylation provide advantages to the cancer cells.	Class A-II	G9
	928 Sequences (65.31%)	Not hypermethylated in hCCLs	1 Sequences (0.07%) hypermethylated in all NTTs (>0.7 in 6/6 samples)	Genes hypermethylated early during hESC differentiation. These genes could be aberrantly hypomethylated in cancer.	-	G10
		Not>0.7 in≥6/21 samples	404 Sequences (28.43%) unmethylated in all NTTs (<0.3 in 6/6 samples)	Genes constitutively hypomethylated	-	G11
		464 Sequences (32.65%)	52 Sequences (3.66%) sometimes unmethylated (≤0.3 signal in ≥1/6 and ≤5/6 samples)	Genes which hypermethylation during hESC differentiation might be important for lineage specification. These genes could be aberrantly hypomethylated in cancer.	-	G12

The classification criteria are described in the Methods section.

Significantly, we found that 34.69% (493/1,421) of the sequences were frequently hypermethylated in hESCs (array signal>0.7 in≥25% (2/8) of the samples). Most of these (79.72%, 393/493) were also frequently hypermethylated in CCLs (array signal>0.7 in≥25% (6/21)) of the samples). Again, many of them (32.32%, 127/393) were unmethylated in at least one of the NTTs analyzed (array signal<0.3 in≥17% (1/6) NTTs) (**Figure 1**, and **Tables 1** and **S1**). In contrast to the Class A cancer hypermethylated genes, those of this group were also frequently hypermethylated in hESCs, so we propose that they can be considered members of a different category of cancer methylated genes, which we have termed Class B cancer hypermethylated genes. Of the 697 sequences frequently hypermethylated in cancer and unmethylated in at least one of the NTTs analyzed, 444 (66.70%) and 127 (18.22%) were respectively classified as Class A and Class B genes (**Figure 1**, and **Tables 1** and **S1**), which indicates, contrary to expectation, that a substantial proportion (around 20%) of cancer methylated genes are also frequently hypermethylated in hESCs.

Intriguingly, not all the genes frequently hypermethylated in CCLs were completely unmethylated in all the NTTs analyzed (**Figure 1**, and **Tables 1** and **S1**). The frequency of hypermethylation in normal tissues is only of moderate importance for the Class A classical cancer methylated genes, as most of them (78.83%; 350/444 sequences) are unmethylated in NPTs. On the other hand, only 20 sequences corresponding to Class B genes were unmethylated in all the NTTs analyzed (**Table 1**). When a gene is methylated in some but not all normal tissues, the methylation is probably involved in the specification of a tissue type during development [12]. When the gene is not hypermethylated in hESCs, tissue-type-dependent selective methylation must occur. In contrast, when the gene is frequently hypermethylated in hESCs, it most probably becomes selectively demethylated upon differentiation as an epigenetic mechanism that is able to facilitate tissue specification. Conversely, when a gene is unmethylated in all normal differentiated cells and hypermethylated in stem cells, the loss of promoter methylation that necessarily occurs during differentiation is more likely to be involved in early differentiation processes than in tissue specification [12]. Thus, we defined two new subcategories for both Class A and B cancer methylated genes: Subcategory I, for genes that are always unmethylated in normal tissues, and subcategory II, for genes that are sometimes methylated in normal tissues (**Figure 1**, and **Tables 1** and **S1**). The percentage of Class A-II and Class B-II genes is quite similar (7.53% and 6.61%) (**Table 1**). However, the percentage of genes in Class A-I (24.63%) is much higher than that in Class B-I (1.41%). The genes in these four categories (A-I, A-II, B-I, and B-II) represent 58.2% of all the sequences present in the methylation arrays. By considering the methylation status of the three groups of samples (hESCs, NPTs, and CCLs) we were able to cluster most of the remaining genes in the array into eight additional categories (**Table 1**), which included, for example, two categories of genes that we define as being constitutively methylated (methylated in hESCs, CCLs, and all NTTs; 11.19%) or constitutively unmethylated (unmethylated in hESCs, CCLs, and all NTTs; 28.43%) genes, respectively. We therefore propose that DNA methylation is not important for the regulation of the genes in these categories. The classification of genes according to their methylation status in hESCs, CCLs, and NTTs, and the interpretation of the biological role of DNA methylation in the genes in each group is summarized in **Table 1**. **Table S1** lists the genes in each group.

It is important to note here that all the previously described percentages refer to the 807 genes included in methylation arrays, whereas the overall percentage of genes in each group might be different if the entire genome were considered. The classification threshold that we employed to identify genes frequently hypermethylated in hESCs (more than 70% of promoter CpG methylation in more than 25% of samples analyzed) is that which is commonly used to define a gene as being frequently hypermethylated in cancer [13]. To assess whether our observations hold true for astringent classification thresholds we reexamined our data in search of: i) sequences hypermethylated in most of the hESCs analyzed (array signal>0.7 in≥75% (6/8) of the hESCs) and, ii) sequences “fully methylated” in some of the hESCs analyzed (array signal>0.8) in≥25% (2/8) of the hESCs) (**Table S2**). We found that 5 B-I and 84 B-II sequences fitted the first criterion, and 13 B-I and 86 B-II sequences fitted the second (**Table S2**), which indicates that our conclusions remain valid even with these stricter classification thresholds.

It has recently been shown that prolonged *in vitro* culture of hESCs is associated with DNA methylation instability [14], [15]. To assess whether promoter hypermethylation of our Class B genes is associated with the *in vitro* culture process, we compared our data with those previously published by Bibikova *et al.* [11]. These authors used the Goldengate methylation platform to compare the methylation status of the 1505 CpG sites contained in the arrays in ten hESC lines at low and high passages. They found that, although methylation changes did occur with passage number, such changes were small compared with the differences among cell types. They found five genes (*ASCL2*, *GALR1*, *MEST*, *NPY,* and *SLC5A8*) to be consistently hypermethylated with the passage number (increase in methylation level>0.34 in at least two cell lines (20%)). Three of those genes (*ASCL2*, *NPY,* and *SLC5A8*) are members of Class B-I but, interestingly, none was a Class B-II gene. These results suggest that prolonged *in vitro* culture was only responsible for the promoter hypermethylation of a small fraction (3/97, 3%) of our Class B genes, and that the effect appeared to be greater in Class B-I genes.

As previously stated, it has recently been proposed that developmental genes are silenced in embryonic stem cells by a Polycomb-dependent bivalent histone-based chromatin mark [4], [5], which is thought to predispose to aberrant DNA promoter hypermethylation of TSGs in cancer [7]–[9]. As we found that a subset of cancer methylated genes can also be methylated in hESCs we wanted to investigate the relationship between promoter hypermethylation and the Polycomb-dependent histone modification pattern in hESCs. To this end, we compared our methylation data for the Class A-I, A-II, B-I, and B-II genes with the previously reported histone modification profile and Polycomb occupancy of the same genes in embryonic stem cells [5], [12], [16] (**Figure 1** and **Table S3**). Consistent with a previous report [9], we found that around 35% of the sequences frequently hypermethylated in cancer and unmethylated in at least one of the NTTs analyzed contained chromatin-repressive marks at their promoters (228-277/697 harbored meK27, and 236/697 contained SUZ12). Intriguingly, comparing our methylation data with those of Mikkelsen *et al.* [16] we observed that the vast majority (96.4%) of genes harboring meK27 also contained meK4 and that only around 30% of the genes frequently hypermethylated in cancer presented the bivalent chromatin domain (meK4/meK27) in hESCs (**Table S3**).

When we compared the chromatin patterns and Polycomb occupancy in the Class A-I, A-II, B-I, and B-II genes we found each group to have a specific chromatin signature (p<0.00001). Class A genes were more enriched in Polycomb and bivalent marks (47.5% and 45.5–57.3% of genes, respectively) than Class B genes (19.7% and 21.4–32.7%, respectively) (p<0.00001) (**Figure 1B** and **Table S4**). The enrichment of the bivalent mark in Class A genes is primarily due to the low levels of this chromatin signature in Class B-II genes (**Table S4**). Indeed, Class B-I genes exhibit similar levels of meK4/meK27 to Class A genes (**Table S4**) (p<0.00001). Interestingly, the Class II genes, were much less frequently occupied by Polycomb proteins and exhibited fewer bivalent marks (33.3% and 26.5–38.8% of genes, respectively) (p<0.00001) than did Class I genes (45.7% and 47.6–59.3%, respectively), (**Figure 1B** and **Table S4**). The lower levels of the bivalent mark in Class II genes were primarily due to the low levels of this chromatin signature in Class B-II genes (**Table S4**). Indeed, Class A-II genes had similar levels of meK4/meK27 to those of Class I genes (**Table S4**) (p<0.00001).

### TSGs repressed by promoter hypermethylation in hESCs

**Figure 2:**
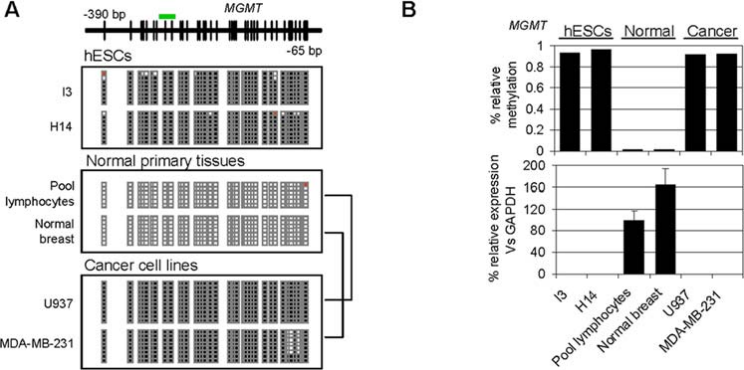
Promoter DNA hypermethylation and repression of *MGMT* in hESCs. (A) Bisulfite genomic sequencing of multiple clones of the MGMT promoter in hESCs (I3, H14), normal primary tissues (Pool lymphocytes, normal breast) and two CCLs of lymphoid and breast origin (U937 and MDA-MB-231, respectively). Black, methylated CpG; white, unmethylated CpG; red, CpG not present. The green bar above the diagram of the MGMT CpG island indicates the location of the probe used in the methylation arrays. (B) Relationship between MGMT promoter hypermethylation and expression in hESC, normal, and cancer samples. The upper panel shows the relative methylation signal obtained with the methylation arrays and the lower panel the expression levels of MGMT mRNA relative to GAPDH.

To test the hypotheses formulated on the basis of the data obtained from the methylation arrays, we focused our attention on four Class B genes (frequently hypermethylated in cancer and hESCs) that were previously widely reported to be genes with tumor suppressor properties and that are frequently hypermethylated in cancer. We selected two (MGMT and SLC5A8) [17], [18] from the Class B-I subcategory (unmethylated in all NTTs) and two (PYCARD and RUNX3) [19], [20] from Class B-II (unmethylated in a number of NTTs). We first employed bisulfite sequencing of multiple clones to determine accurately the promoter DNA methylation status of these genes in hESCs and normal tissues (**Figure 2A**, and **Figures S2, S3, S4, S5**). In all cases, bisulfite sequencing data corroborated the results obtained from the arrays and showed that the hypermethylation observed in hESCs affected the majority of the CpGs surrounding the transcriptional start-site of the selected genes. MGMT and SLC5A8 (Class B-I) presented dense promoter hypermethylation in hESCs but not in normal differentiated tissues (**Figure 2A**, and **Figures S2, S3**) whilst Class B-II genes were frequently hypermethylated in hESCs and sometimes in normal tissues (**Figures S4, S5**). To understand better the role in promoter hypermethylation of our selected TSGs in hESCs and NTTs, we used q-RT-PCR to measure their expression in both sample groups (**Figure 2B**, and **Figures S2, S3, S4, S5**). We found that promoter hypermethylation was always associated with gene repression, but its absence in somatic primary tissues did not necessarily involve the upregulation of the gene. For example, whilst *SLC5A8* was hypomethylated in all the normal tissues analyzed (**Figure S3A,B**), it was only overexpressed in brain, liver, and colon (**Figure S3C**).

### Loss of promoter hypermethylation and gene activation during *in vitro* differentiation of hESCs

**Figure 3:**
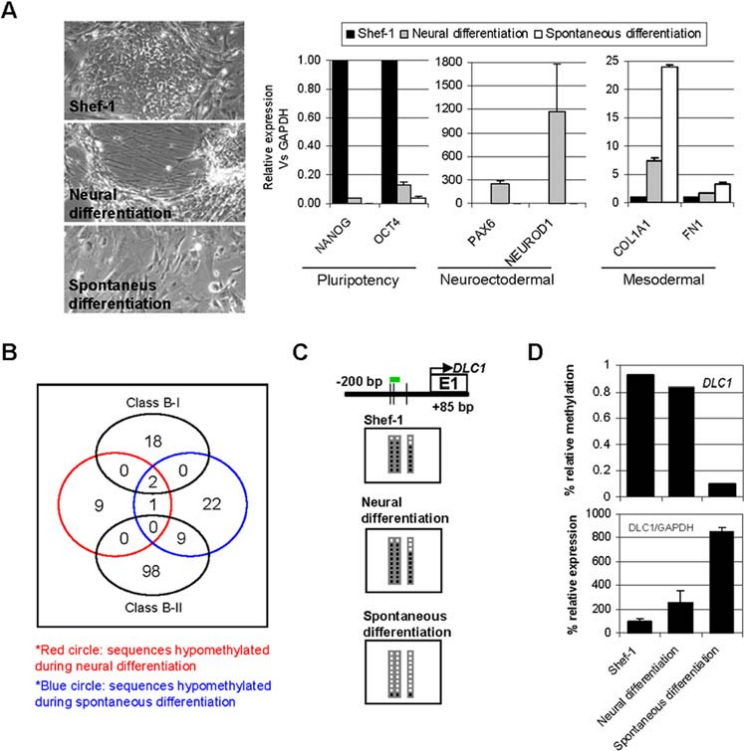
Loss of promoter DNA methylation during *in vitro* differentiation of hESCs. (A) Left-hand images, Shef-1 stem cell line (upper) and the same cells after neural differentiation (middle) and spontaneous differentiation to fibroblast-like cells (lower). The right-hand panels show the relative mRNA levels of pluripotency (NANOG, OCT4), neuroectodermal (PAX6, NEUROD1), and mesodermal (COL1A1, FN1) markers before and after Shef-1 differentiation. (B) Number of sequences hypomethylated during Shef-1 neural (red circle) and spontaneous (blue circle) differentiation, and their overlap with Class B-I and Class B-II genes (black circles). (C) Bisulfite genomic sequencing of multiple clones of the DLC1 promoter in Shef-1 stem cell line (upper) and the same cells after neural differentiation (middle) and spontaneous differentiation to fibroblast-like cells (lower). The color code is as for Figure 2. (D) Relationship between DLC1 promoter hypermethylation and expression during differentiation of Shef-1 cells. The upper panel shows the relative methylation signal obtained with the methylation arrays and the lower panel the expression levels of DLC1 mRNA relative to GAPDH.

To demonstrate further that the differentiation of hESCs is associated with less DNA methylation at the promoter region of certain genes, we induced the *in vitro* differentiation of the hESC line Shef-1 in two cell lineages (fibroblast-like cells and neural precursors) (**Figure 3A**). We assessed the lineage specification using previously published markers [21] (**Figure 3A**, right-hand panels) and then used methylation arrays to identify genes that became hypomethylated during differentiation. We found that 12.98% (37/285) of the genes hypermethylated in Shef-1 (which were not methylated in all the NTTs analyzed) become unmethylated during *in vitro* differentiation. Of these, 12 genes become unmethylated during neuron differentiation and 25 during spontaneous differentiation (**Table S5**). Three of these genes were common to both groups (**Figure 3B**) and two of them belonged to Class B-II. It is of particular note that, whilst 9/25 of the genes unmethylated during spontaneous differentiation were of Class B-II, none of the 12 genes unmethylated during neuron differentiation belonged to this category.

To demonstrate that some TSGs that are frequently hypermethylated in cancer and hESCs can lose methylation during differentiation, we focused our attention on DLC1. We chose this gene because the methylation arrays had shown that it lost promoter methylation during spontaneous differentiation of Shef-1, and because it is known to be a TSG that is frequently hypermethylated in cancer (**Figure S6**) [22], [23]. Bisulfite sequencing of multiple clones corroborated the results obtained with the methylation arrays and showed that DLC1 promoter is hypermethylated in Shef-1 and becomes unmethylated during spontaneous, but not neural, differentiation (**Figure 3C**). In q-RT-PCR experiments DLC1 was poorly expressed in the Shef-1 cell line and became overexpressed during spontaneous, but not neural, differentiation (**Figure 3D**).

### TSGs repressed by promoter hypermethylation in hematopoietic stem cell progenitors

**Figure 4:**
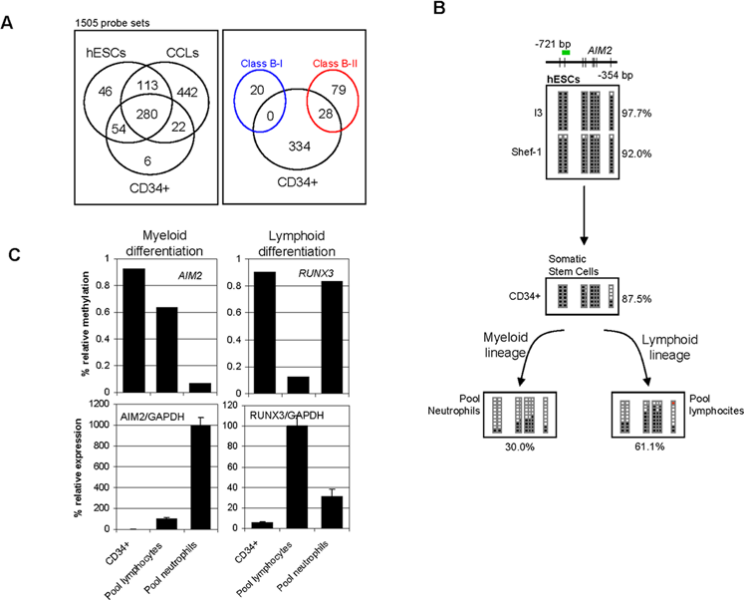
Cancer genes hypermethylated in somatic stem cells. (A) The left-hand panel shows the numbers of sequences that are hypermethylated in the somatic stem cells CD34+, and hypermethylated in hESCs and CCLs. Note that most of the sequences hypermethylated in somatic stem cells are also hypermethylated in embryonic stem cells. The right-hand panel shows the number of sequences hypermethylated in CD34+ cells (black circle) classified as Class B-II genes (red circle). Sequences hypermethylated in CD34+ cells were never classified as Class B-I genes (blue circle). (B) Bisulfite genomic sequencing of multiple clones of the AIM2 promoter in Shef-1 and I3 stem cell lines (upper), CD34+ hematopoietic stem cell progenitors (middle), and terminally differentiated hematopoietic cells (peripheral lymphocytes and neutrophils). The color code is as for Figure 2. (C) Relationship between *AIM2* and *RUNX3* promoter hypermethylation and expression in CD34+ somatic hematopoietic stem cell progenitors and terminally differentiated hematopoietic cells (peripheral lymphocytes and neutrophils). The upper panel shows the relative methylation signal obtained with the methylation arrays and the lower panel the expression levels of *AIM2* and *RUNX3* mRNAs relative to GAPDH.

Having demonstrated that some cancer genes are hypermethylated and repressed in hESCs and that they can lose methylation during *in vitro* differentiation of hESCs, we investigated whether this phenomenon is restricted to embryonic development or, conversely, is an epigenetic mechanism associated with stemness status regardless of the ontogenetic stage of the cell. We used methylation arrays to identify genes hypermethylated in CD34+ somatic stem cell progenitors (**Figure S7**) compared with peripheral blood lymphocytes and neutrophils, two types of adult primary cells derived from the CD34+ hematopoietic progenitors. We identified 362 significantly hypermethylated sequences in the CD34+ cells (array signal>0.7) (**Table S6**). The vast majority of these sequences (92.27%, 334/362) were also methylated in hESCs and most were frequently hypermethylated in CCLs (83.43%, 302/362) (**Figure 4A**, and **Table S6**). These results suggest that the hypermethylation of cancer genes can occur in stem cells regardless of the ontogenetic stage (embryo vs. adult). We next identified nine sequences that were significantly hypermethylated in CD34+ cells relative to peripheral lymphocytes, and 16 sequences that were hypermethylated in these progenitor cells relative to neutrophils (**Table S7**). Most of the sequences identified were also frequently hypermethylated in hESCs (8/9 for lymphocytes and 14/16 for neutrophils) and CCLs (6/9 for lymphocytes and 13/16 for neutrophils). In addition, there were no sequences common to lymphocytes and neutrophils and most of them were sometimes hypermethylated in NPTs. Interestingly, 28 of these sequences were previously classified as Class B-II genes whilst none of them was from Class B-I (**Figure 4A**).

Finally, to demonstrate that some cancer methylated genes are also frequently methylated in somatic progenitor stem cells and that their methylation is important for lineage specification, we considered two genes: *RUNX3* and *AIM2*. We selected *RUNX3* because, in agreement with previously published data [24], our methylation arrays showed that, relative to CD34+ cells, *RUNX3* was hypomethylated in peripheral lymphocytes but not in peripheral neutrophils. *AIM2* was selected because it was previously thought to be a TSG that is frequently hypermethylated in cancer (**Figure S8**) [25] and because, unlike *RUNX3*, it becomes unmethylated specifically in the myeloid lineage (**Figure 4B,C**). The bisulfite sequencing data confirmed the results obtained with the arrays, showing that the CD34+ cells and the peripheral lymphocytes were densely methylated at the promoter of *AIM2* gene whilst the peripheral neutrophils were almost unmethylated (**Figure 4B**). To determine the role of promoter hypermethylation of *AIM2* and *RUNX3* in hematopoietic differentiation, we used q-RT-PCR to analyze their expression in our groups of samples (**Figure 4C**). We found that promoter hypermethylation was always associated with gene repression in both genes and that loss of promoter methylation in *AIM2* and *RUNX3* in peripheral lymphocytes and neutrophils, respectively, was associated with their reexpression (**Figure 4C**).

## Discussion

Aberrant promoter hypermethylation of TSGs and differentiation factors is a central epigenetic alteration in cancer [26], [27]. The genes undergoing such alterations in cancer have been reported to be repressed in hESCs by the establishment of “bivalent chromatin domains” consisting of activating (H3 lysine 27 methylation) and repressing (H3 lysine 4 methylation) histone marks that keep them poised for activation but, at the same time, predisposes them to aberrant promoter hypermethylation in adult cancers [7]–[9]. Herein we demonstrate that another level of complexity exists whereby some of the genes frequently aberrantly hypermethylated in cancer are also frequently hypermethylated in hESCs. Our results suggest that, as was previously proposed for mouse cells [28], promoter DNA methylation can be an important factor in gene regulation in hESCs.

On the basis of the methylation status in hESCs we established two categories of cancer methylated genes: Class A genes, which are frequently unmethylated in hESCs, and Class B genes, which are frequently hypermethylated in hESCs. As we unexpectedly found that a substantial proportion of the genes included in both groups were also frequently hypermethylated in normal differentiated tissues, we established two new subcategories of cancer methylated genes: subcategory I, for genes that are mostly unmethylated in normal tissues, and subcategory II, for genes that are sometimes hypermethylated in normal tissues. The biological interpretation of aberrant methylation within Classes A and B cancer methylated genes and their two subcategories is completely different. Class A-I genes are frequently hypermethylated in cancer but not in normal tissues or hESCs. These genes are not supposed to be regulated by DNA methylation during normal development and thus the hypermethylation in cancer should always be interpreted as an aberrant process. Class A-II genes are frequently methylated in CCLs and sometimes in normal tissues, but rarely in hESCs. Methylation of these genes may be important for lineage specification and should be considered aberrant in cancer when it occurs in a tumor type in whose corresponding normal tissue it is not hypermethylated. Class B-I genes (excluding *ASCL2*, *NPY,* and *SLC5A8* genes, whose promoter DNA hypermethylation in hESC lines could be due to the *in vitro* culture process [11]) are frequently hypermethylated in hESCs and cancer cells lines but never in normal tissues, which suggests that the loss of methylation at the promoters of these genes might be an important influence on the loss of pluripotency during development. Their hypermethylation in cancer should always be considered as aberrant. Class B-II genes are frequently hypermethylated in hESCs and cancer cells, but, as they are also sometimes methylated in normal tissues, their hypermethylation in cancer may only be considered aberrant in tumor types in whose normal counterparts they are completely unmethylated. Apart from this, the fact that not all the genes frequently hypermethylated in cancer were completely unmethylated in all the normal tissues analyzed is a highly important finding in cancer epigenetics [26] because the promoter hypermethylation of a gene in a particular tumor type should not be considered aberrant when the promoter of this gene is equally hypermethylated in its non-tumorigenic counterpart.

We found the percentage of Class A-II and Class B-II genes to be quite similar (7.53% and 6.61%), which suggests that the probability of aberrant hypermethylation or improper loss of methylation is similar in genes in which hypermethylation or loss of methylation, respectively, is necessary for lineage specification. However, the percentage of genes in Class A-I is much higher than that in Class B-I (24.63% and 1.41%), implying that it is much easier for a gene that is not naturally regulated by DNA methylation to become aberrantly hypermethylated than for there to be loss of methylation of developmental genes during hESC differentiation.

Comparing our DNA methylation data with those previously published on the histone modification profile and Polycomb occupancy of the same genes in embryonic stem cells [5], [12], [16] we found that the vast majority of genes harboring meK27 also contained meK4. This concurs with the observation that most of the genes repressed in stem cells by Polycomb-repressive marks also contain histone-active marks [4]. However, as only around one-third of the genes frequently hypermethylated in cancer presented the bivalent chromatin domain (meK4/meK27) in hESCs, our results suggest that this chromatin signature in cancer methylated genes could be less abundant in hESCs than previously expected. However, we must recognize that our study is limited to the set of 807 genes selected for inclusion in the methylation arrays, and that the overall pattern needs to be determined in genome-wide DNA methylation studies, to establish how general our observations are.

Within our four categories of genes we found those of Class A to be more enriched in Polycomb and bivalent marks than Class B genes, which suggests that the previously described scenario involving bivalent chromatin domains and Polycomb occupancy of cancer methylated genes in embryonic stem cells [7]–[9] could be more frequent in Class A than in Class B genes. Interestingly, the Class II genes, which we previously suggested were involved in lineage specification because their promoter methylation was tissue-dependent, were much less frequently occupied by Polycomb proteins and exhibited fewer bivalent marks than did Class I genes, which we previously believed to be involved in early differentiation processes. The lower levels of the bivalent mark in Class II genes were primarily due to the low levels of this chromatin signature in Class B-II genes, implying that this subcategory of genes might be relevant to lineage specification. Zhao *et al.* [12] proposed that genes harboring meK4/meK27 bivalent marks were primarily involved in early differentiation, whilst those without either mark were involved in lineage specification. As we observed that Class I genes were enriched in the bivalent mark and Class B-II genes were frequently depleted of both marks, the data of Zhao *et al.* [12] support our hypothesis that Class I genes are primarily involved in early differentiation processes whilst Class B-II (excluding those whose promoter DNA hypermethylation depends on prolonged *in vitro* culture) are more associated with lineage specification. This hypothesis is further supported by the fact that the gene ontology classification of the two categories shows that Class I genes are associated with biological processes involved in early differentiation, whilst most Class B-II genes are associated with those linked to lineage specification (**Table S8**).

To investigate further the role of promoter DNA methylation of genes aberrantly hypermethylated in cancer in hESCs, we compared the DNA methylation and expression status of four of the genes identified in the methylation arrays (MGMT and SLC5A8 [17], [18] from Class B-I, and PYCARD and RUNX3 [19], [20] from Class B-II). We found that promoter hypermethylation was always associated with gene repression, but its absence in somatic primary tissues did not necessarily involve the upregulation of the gene, as was the case for *SLC5A8*, in which there was no overexpression upon loss of promoter methylation in peripheral lymphocytes. In view of this, we hypothesized that promoter hypermethylation of the Class B cancer methylated genes in hESCs (excluding those whose promoter DNA hypermethylation depends on prolonged *in vitro* culture [11]) can be a natural process employed by stem cells to ensure the silencing of genes whose expression is associated with stemness status. When stem cells differentiate, these genes may lose their promoter hypermethylation, which could allow the genes to become overexpressed. However, this does not imply that the gene has become immediately activated, as demonstrated by the fact that some of these genes were still repressed in differentiated tissues. The loss of promoter methylation during differentiation could merely keep these genes poised for activation until they are later required by the somatic cells. The lack of hypomethylation-associated activation in some mature tissues suggests that signals other than the lack of methylation are necessary for these genes to be activated.

By forcing the *in vitro* differentiation of the hESC line Shef-1 we identified 12 and 25 genes that become unmethylated respectively during neuron and spontaneous differentiation. Interestingly, three of these genes were common to both groups, suggesting that they may be involved in early differentiation processes. This implication is supported by the fact that two of the genes were from Class B-I, which we previously proposed were involved in early development. Whilst the majority of genes unmethylated during spontaneous differentiation were of Class B-II, none of the genes unmethylated during neuron differentiation belonged to this category. This may largely be explained by the fact that the ten genes that were unmethylated during neuron differentiation and did not belong to Class I or II were not classified as “unmethylated in at least one normal tissue type analyzed” because their intermediate levels of methylation in normal tissues did not allowed their classification into any of the four categories described. Moreover, there were many more hypomethylated genes (relative to Shef-1) in normal brain than in neural Shef-1-derived cells. The observations suggest two things: i) the *in vitro* differentiation of hESCs does not reproduce all the epigenetic features present during *in vivo* differentiation, and ii), the unguided spontaneous differentiation of our hESC achieves more epigenetic hits *in vivo* than does neural differentiation.

One of the genes that we identified using this approach is *DLC1*,. a TSG frequently inactivated by promoter hypermethylation in cancer cells [22], [23]. We found that *DLC1* becomes unmethylated and overexpressed specifically during spontaneous, but not neural, differentiation of the Shef-1 cell line, which suggests that loss of DNA methylation-dependent expression of this gene might be involved in lineage specification. This is supported by the essential role of *DLC1* during embryonic development, whereby *DLC1*-deficient mice are unviable [29].

Finally, we wondered whether the methylation-dependent repression of cancer genes in hESCs is a molecular process associated with embryonic development or if, by contrast, it is an epigenetic mechanism involved in the maintenance of stemness status. The fact that the CD34+ somatic stem cell progenitors featured numerous genes frequently hypermethylated in cancer that are repressed by promoter hypermethylation suggests that, at least for these genes, the process could be associated with stemness status regardless of the ontogenetic stage of the cell, rather than being an event restricted to embryonic development. Since CD34+ cells are primary non-cultured cells, we can also discount the possibility that *in vitro* culture of the hESCs is largely responsible for hypermethylation, which accords with previously published findings [11], [30], [31].

**Figure 5:**
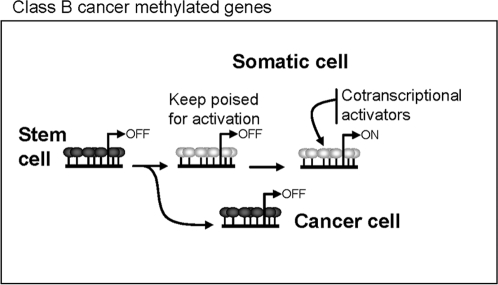
Proposed model of aberrant methylation in cancer for genes frequently hypermethylated in stem cells *in vivo.* The loss of promoter hypermethylation might be necessary for overexpression of a subset of Class B genes during differentiation. The aberrant process in cancer for these genes should be understood as a defect in establishing an unmethylated promoter during differentiation, rather than as an anomalous process of *de novo* hypermethylation.

By comparing the DNA methylation status of CD34+ progenitor cells with those of two types of primary cells that are terminally differentiated from the former (peripheral blood lymphocytes and neutrophils) we identified several genes that lost methylation specifically in just one of lineages. This, in conjunction with knowledge that most of the sequences identified were sometimes hypermethylated in NPTs and most were previously classified as Class B-II genes (those whose regulation by methylation is important for lineage specification and that present aberrant methylation in cancer), suggests that the genes hypermethylated in CD34+ progenitor cells that become unmethylated during differentiation are those primarily involved in lineage specification. That none of the sequences identified in the CD34+ progenitor cells was from Class B-I may well be because the CD34+ cells are not the primary hematopoietic progenitor cells and because Class B-I genes lose methylation in the transition from earlier progenitor stem cells to CD34+ cells. This explanation is consistent with the putative role of these genes in early development [12] but needs further investigation. Moreover, from our point of view, the fact that some cancer methylated genes are also frequently hypermethylated in adult stem cells is particularly important to our understanding of aberrant methylation in cancer. In the context of the hypothesis of the stem cell origin of cancer [32], [33], our results suggest that, for TSGs hypermethylated in stem cells *in vivo*, the aberrant process in cancer could be understood as a defect in establishing an unmethylated promoter during differentiation, rather than as an anomalous process of *de novo* hypermethylation (**Figure 5**).

Using the above approach, we identified two genes, *AIM2* and *RUNX3,* that were hypermethylated and repressed in CD34+ hematopoietic progenitor cells and that became unmethylated and overexpressed in myeloid and lymphoid lineages, respectively. Both genes are aberrantly hypermethylated in cancer [20], [25], which indicates that genes frequently hypermethylated in cancer can be naturally repressed by promoter methylation not only in hESCs but also in somatic stem cells. Moreover, the lineage-specific loss of methylation and upregulation of the two *genes* suggests that their expression might be important in lineage specification during hematopoietic differentiation and, more importantly, that the process can be regulated by DNA methylation. *RUNX3* is a well-known transcription factor that regulates lineage-specific gene expression in developmental processes [34]. Our observation that *RUNX3* loses methylation and becomes expressed during lymphoid, but not myeloid, development is consistent with previous studies showing the necessity of *RUNX3* for T cell development during thymopoiesis [35], [36], and that RUNX3 knockout mice have T cell phenotypes [20], [37]. Finally, considering all the available evidence and following similar reasoning to before, the aberrant hypermethylation of *RUNX3* observed in the lymphoid human CCL Raji should be understood as the failure of CD34+ cells to lose the promoter methylation necessary to reactivate the gene during hematopoietic differentiation.

The results presented here are important for four reasons: i) we unexpectedly found a subset of cancer methylated genes that are also frequently methylated in hESCs; ii) the pattern of expression of these genes implies that DNA methylation might have an important role in the control of their expression in hESCs; iii) determining DNA methylation status in hESCs allowed us to define two categories of cancer methylated genes: Class A, containing genes that are never hypermethylated in hESCs, and Class B, containing genes that are frequently hypermethylated in hESCs; and, probably most important, iv) the hypermethylation of some Class B genes in adult stem cells *in vivo* suggests that, for this group of genes, the aberrant methylation in cancer can be understood as a defect in establishing an unmethylated promoter during differentiation, rather than as an anomalous process of *de novo* hypermethylation.

## Materials and Methods

### Stem cell lines

Cell pellets and/or DNA/RNA were obtained from the following laboratories: Shef-1 (Servicio de Inmunologia, HUCA, Oviedo, Spain), Shef-4, Shef-5, Shef-7, H7, H14 (CSCB, University of Sheffield, Sheffield, UK), H181 (CABIMER, Seville, Spain), I3 (Institute of Reconstructive Neurobiology, University of Bonn, Germany), and cultured and passaged following established protocols by each laboratory. The laboratories that were involved in the establishment and maintenance of these cell lines are members of the European project ESTOOLS (LSHG-CT-2006-018739). The laboratories participating in ESTOOLS only use embryonic stem cell lines derived from IVF embryos that will not be transferred into the womb. These embryos were donated for research according to the legal requirements of the country of origin. All donors gave their written informed consent. Profiling epigenetic regulation in hESCs is one of the research objectives of the ESTOOLS research program, which is supervised by the ethics advisory panel of the ESTOOLS project. The cell lines were established from different embryos and were maintained under different conditions, thereby ensuring the independence of our results for type of line and culture conditions. *In vitro* differentiation of the Shef-1 cell line was achieved as previously described [38].

Primary CD34+ hematopoietic somatic stem cells were purified from cord blood (CB) samples obtained from healthy newborns upon progenitor's informed consent. CB harvesting procedures and informed consents were approved by the Local Hospital Ethics Board. Mononuclear cells were isolated using Ficoll-Hypaque (Amersham Biosciences, Baie d'Urfé, Quebec, Ontario, Canada). CD34+ cells were purified by positive selection using anti-CD34 microbeads (Miltenyi Biotech, Madrid, Spain). Immunomagnetic CD34+ cell-containing cell suspensions were passed through Pro-MACS immunomagnetic columns (Miltenyi Biotech). The flow-through contained the purified CD34+ fraction. The purity was 80% ± 12% (n = 2) (**Figure S7**), as measured by flow cytometry (FACSCanto, Becton Dickinson, Palo Alto, CA) using a fluorochrome-conjugated anti-CD34 antibody (BD).

### Cancer cell lines and primary tissues

MDA-MB-231, Hela, CasKi, SiHa, HCC1937, BT-474, LoVo, HCT115, DLD1, Co115, HT29, SW48, HCT116, RKO, U937, HL60, AKATA, Raji, Ramos, Karpas, and Farage (ATCC) cell lines were maintained in DMEM medium supplemented with 10% FBS and grown at 37°C under 5% CO_2_.

Lymphocytes and neutrophils were separated from peripheral blood of healthy volunteers, by centrifugation, using Histopaque®-1077 (SIGMA). Lymphocyte-enriched fractions were obtained by collecting the upper pillow of mononuclear cells and granulocytes (mainly neutrophils) following hemolysis of the remaining pellet. RNA from breast, liver, heart, muscle, lung, colon, and lymph node samples were obtained from Ambion (Austin, TX). DNA from breast, heart, brain, and muscle was obtained from Biochain (Hayward, CA). The subjects who participated in this study gave written consent to being subjected to the procedures.

### DNA methylation profiling using bead arrays

Methylation was assessed at 1,505 CpG sites using Illumina Goldengate Methylation Arrays©, as described in Bibikova *et al.* [11]. The amount of bisulfite-modified target DNA that hybridizes to each spot of the Illumina chip was quantified and standardized over a range from 0.0 to 1.0 (effectively 0% and 100% likelihood of gene promoter hypermethylation, respectively). In this work, all sequences with at least 70% likelihood of being hypermethylated (hybridization signal≥0.7) were considered hypermethylated for each specific sample, whereas sequences whose equivalent signal was below 30% (hybridization signal<0.3) were considered unhypermethylated.

To identify gene promoters that could be hypermethylated in a significant number of samples of a particular group (human embryonic stem cells, normal tissue types, and CCLs), we selected all sequences whose hybridization signal was ≥0.7 in at least 25% of the samples of each group. In general, sequences were classified by the following stepwise algorithm: First, sequences were classified according to the percentage of hESCs hypermethylated in each specific probe set. Therefore, sequences that were hypermethylated in ≥25% and <25% of samples were considered to hypermethylated and unhypermethylated, respectively. Sequences were then tested for hypermethylation in hCCLs and classified according to the percentage (≥25% or <25%) of hypermethylated samples in each probe set. Finally, the percentages of normal tissue types that were hypermethylated in each probe set were calculated, and sequences were classified as hypermethylated in all normal tissue types (100% of samples with signal ≥0.7), unmethylated in all normal tissue types (100% of samples with signal <0.3) or unmethylated in some of the samples but not in all samples (signal<0.3 in at least one, but not all, samples). This algorithm allowed most sequences in the array to be assigned to one of the 12 groups described in **Table 1**.

We next determined whether any of the groups was significantly enriched in a specific type of histone modification. For this reason, all sequences were classified according to publicly available data on histone-modification and Polycomb occupancy [5], [12], [16]. A chi-square test was performed to identify significant differences in frequencies between the groups of sequences. Up to 27 tests were conducted so Bonferroni-adjusted, two-tailed probabilities of <0.0018 (0.05/27) were considered significant.

### Bisulfite sequencing of multiple clones

DNA methylation was determined by PCR analysis after bisulfite modification of the DNA. Bisulfite genomic sequencing was carried out as previously described [39]. A minimum of six colonies of each sequence and sample were automatically sequenced to determine their degree of methylation. Bisulfite genomic-sequencing primers were designed using Methyl Primer Express Software® (Applied Biosystems). Primer sequences are shown in **Table S9**.

### RNA purification and real-time RT-PCR analysis

RNA was isolated with TRIzol Reagent (Invitrogen) according to the manufacturer's instructions. For RT-PCR, 1 μg of total RNA was reverse-transcribed using the High Capacity cDNA Reverse Transcription Kit (Applied Biosystems). Quantitative real-time RT-PCR was performed using TaqMan® Gene Expression Assays and the ABI PRISM® 7900 sequence-detection system (Applied Biosystems). Data are expressed as means ± SD of three replicates of each experiment.

## Supporting Information

Figure S1 Unsupervised cluster analysis of human embryonic stem cells (hESCs), human cancer cell lines (CCLs), and normal primary tissues based on correlation of methylation profiles of 1,421 sequences. The methylation levels vary from fully methylated (red) to fully unmethylated (white) sequences. The final two rows correspond to in vitro-methylated DNA (IVD), used as a positive control for methylation.
(0.20 MB PDF)

Figure S2 Methylation status of MGMT in hESCs, normal tissues, and CCLs. (A) Methylation profiles of MGMT gene obtained by Illumina arrays and expressed as relative methylation from fully unmethylated (0) to fully methylated (1). (B) Bisulfite genomic sequencing of multiple clones of the MGMT promoter in hESCs and normal primary tissues. Color code as for Fig. 1. (C) Relative expression of MGMT in hESCs and normal tissue. qPCR data are normalized with respect to GAPDH expression and presented as the percentage relative to normal lymphocytes.
(0.04 MB PDF)

Figure S3 Hypermethylation of SLC5A8 in hESCs. (A) Methylation profiles of SLC5A8 gene obtained by Illumina arrays and expressed as relative methylation, from fully unmethylated (0) to fully methylated (1). (B) Bisulfite genomic sequencing of multiple clones of the SLC5A8 promoter in hESCs and normal primary tissues. Color code as for Fig. 1. (C) Relative expression of SLC5A8 in hESCs and normal tissue. qPCR data are normalized with respect to GAPDH expression and are presented as the percentage relative to normal lymphocytes.
(0.03 MB PDF)

Figure S4 Hypermethylation of PYCARD in hESCs. (A) Methylation profiles of PYCARD gene obtained by Illumina arrays and expressed as relative methylation from fully unmethylated (0) to fully methylated (1). (B) Bisulfite genomic sequencing of multiple clones of the PYCARD promoter in hESCs and normal primary tissues. Color code as for Fig. 1. (C) Relative expression of PYCARD in hESCs and normal tissue. qPCR data are normalized with respect to GAPDH expression and are presented as the percentage relative to normal lymphocytes.
(0.03 MB PDF)

Figure S5 Hypermethylation of RUNX3 in hESCs. (A) Methylation profiles of RUNX3 gene obtained by Illumina arrays and expressed as relative methylation from fully unmethylated (0) to fully methylated (1). Red arrow indicates methylation levels in normal lymphocytes purified from blood. (B) Bisulfite genomic sequencing of multiple clones of the RUNX3 promoter in hESCs and normal primary tissues. Color code as for Fig. 1. (C) Relative expression of RUNX3 in hESCs and normal tissue. qPCR data are normalized with respect to GAPDH expression and are presented as the percentage relative to normal lymphocytes.
(0.02 MB PDF)

Figure S6 Hypermethylation of DLC1 in hESCs. Methylation profiles of DLC1 gene obtained by Illumina arrays and expressed as relative methylation, from fully unmethylated (0) to fully methylated (1).
(0.02 MB PDF)

Figure S7 Flow cytometry analysis of the purity of CD34+ cells after purification by positive selection using anti-CD34 microbeads. Detection signals were obtained using a fluorochrome-conjugated anti-CD34 antibody (BD). Purity was 80% ± 12% (n = 2).
(0.01 MB PDF)

Figure S8 Hypermethylation of AIM2 in hESCs. Methylation profiles of AIM2 gene obtained by Illumina arrays and expressed as relative methylation, from fully unmethylated (0) to fully methylated (1).
(0.02 MB PDF)

Table S1 List of genes belonging to each group defined in Supplementary Table 1.
(1.20 MB XLS)

Table S2 List of genes identified as being hypermethylated in hESCs using different classification thresholds than that used in Table S1.
(0.32 MB XLS)

Table S3 Methylation data, histone marks, and Polycomb occupancy for genes in the four main categories: A-I, A-II, B-I, and B-II. Raw data from the methylation array for each sample are included. The final three columns summarize information about histone marks and Polycomb occupation published elsewhere. In the HK4/K27 methylation column, K4 stands for 3me-lysine 4 of histone H3, while K27 stands for 3me-lysine 27 of histone H3. In the Polycomb occupation column (+) and (−) respectively refer to the presence and absence of the protein SUZ12.
(0.89 MB XLS)

Table S4 Histone marks and Polycomb occupation in Class A-I, A-II, B-I, and B-II genes. The first table shows each group separately, the second shows Group A vs. group B, and the third Class I vs. Class II genes. The number of genes is presented with the probability of each modification on the right and the percentage on the left.
(0.04 MB XLS)

Table S5 List of genes that are hypomethylated during in vitro differentiation of the embryonic stem cell line Shef-1. Methylation levels from the Illumina array are reported.
(0.02 MB XLS)

Table S6 Genes that are hypermethylated in CD34+ hematopoietic stem cell progenitors. Methylation levels from the Illumina array are reported.
(0.39 MB XLS)

Table S7 Genes that are hypomethylated in peripheral blood lymphocytes and neutrophils relative to CD34+ hematopoietic stem cell progenitors.
(0.02 MB XLS)

Table S8 Summary of the gene ontology GO terms associated with the genes of Classes A-I, A-II, B-I, and B-II. The analysis was done using the web tool of the PANTHER database. Corresponding probabilities of each term and the chromatin-associated gene function (right), based on Zhao et al. (2007), are presented.
(0.02 MB XLS)

Table S9 Primers used for bisulfite sequencing.
(0.02 MB XLS)
