# Supplementary figures and images for: Cancer Genes Hypermethylated in Human Embryonic Stem Cells
Source: PLoS One. 2008 Sep 29;3(9):e3294. doi: 10.1371/journal.pone.0003294 (PMC2546447; doi:10.1371/journal.pone.0003294)

Figure S1

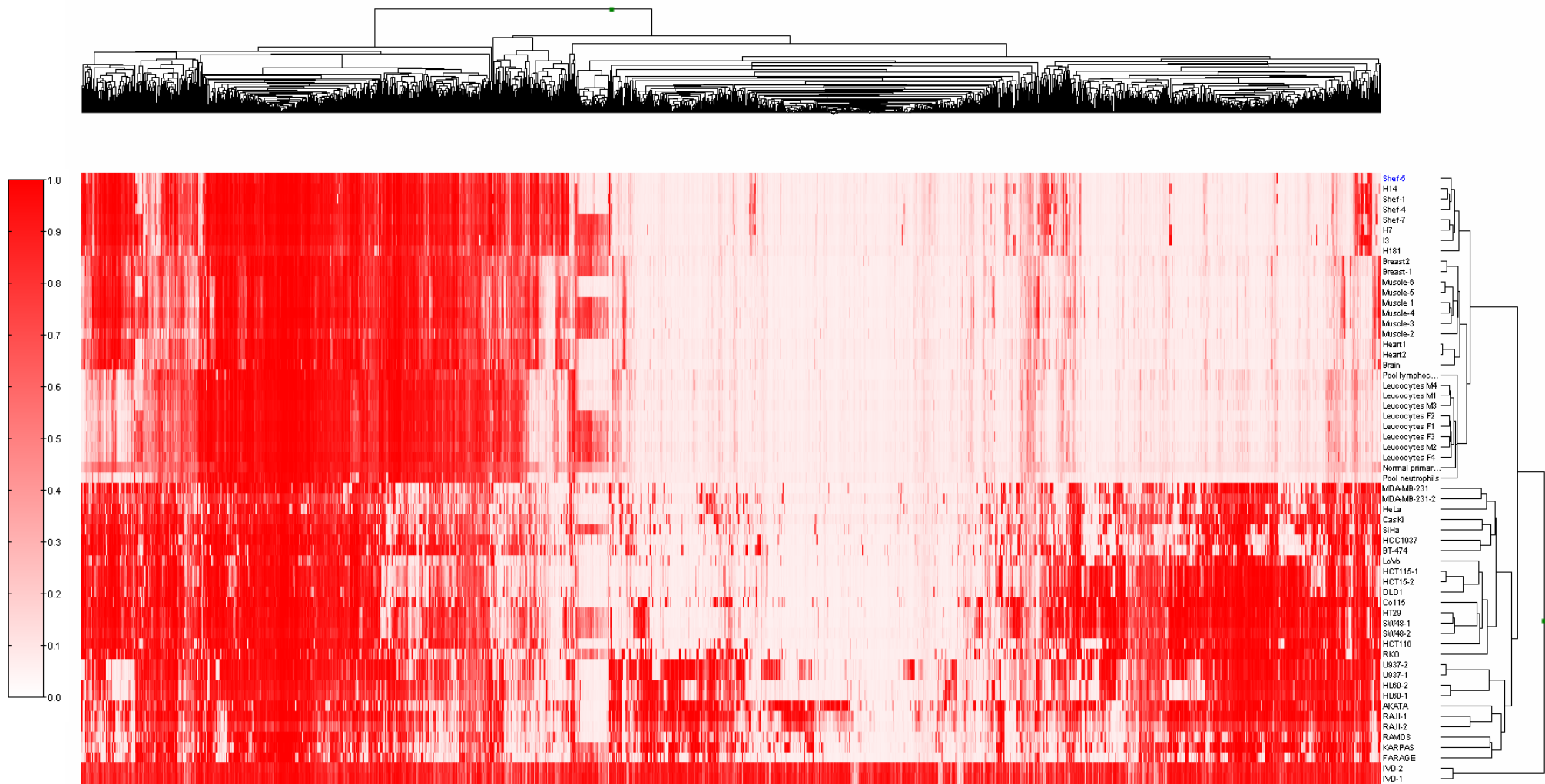

Supplement: Figure S1 — Unsupervised cluster analysis of human embryonic stem cells (hESCs), human cancer cell lines (CCLs), and normal primary tissues based on correlation of methylation profiles of 1,421 sequences. The methylation levels vary from fully methylated (red) to fully unmethylated (white) sequences. The final two rows correspond to in vitro-methylated DNA (IVD), used as a positive control for methylation. (0.20 MB PDF) [file pone.0003294.s001.pdf]

Figure S2

Class B-I: *MGMT*

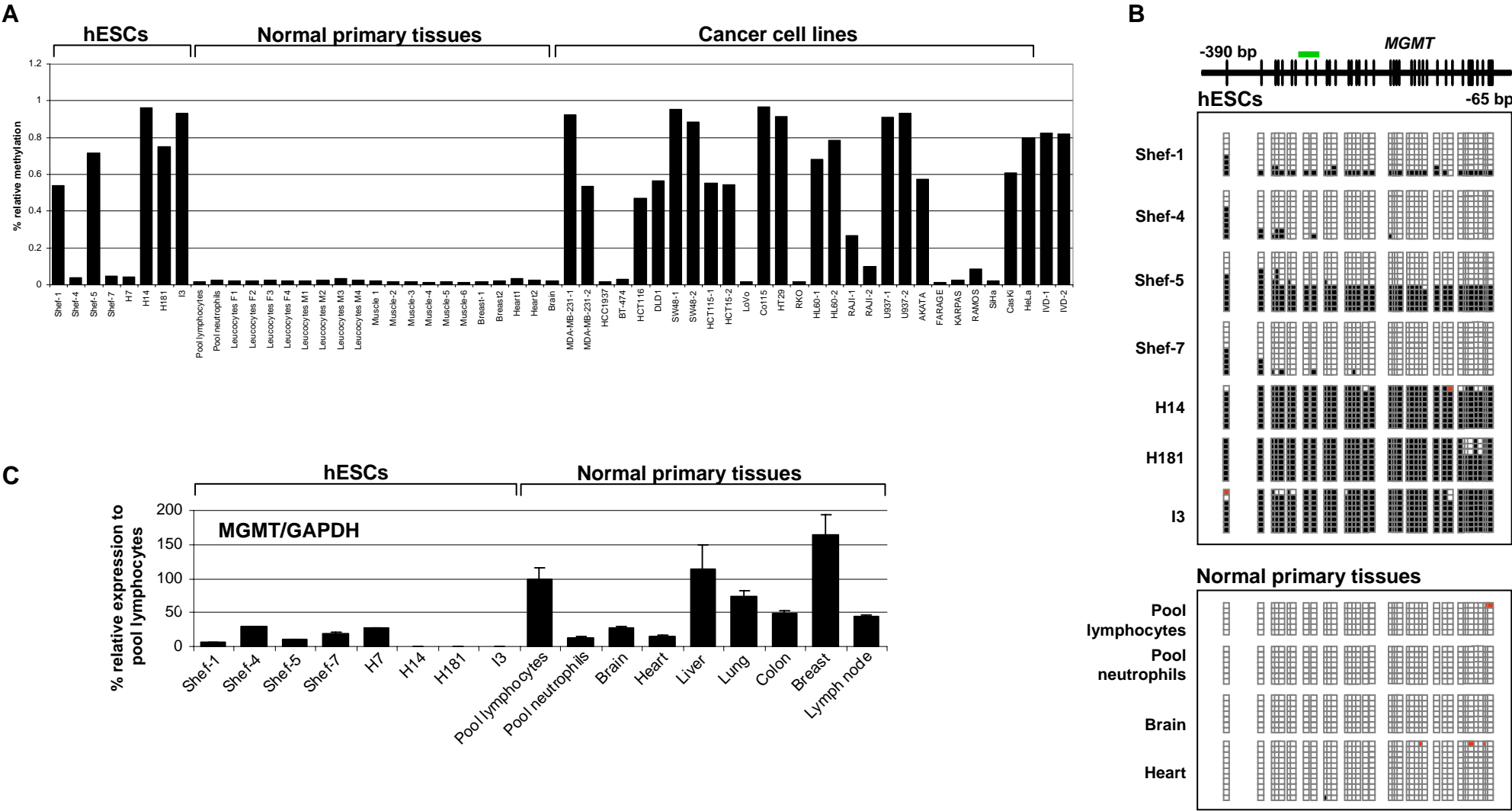

Supplement: Figure S2 — Methylation status of MGMT in hESCs, normal tissues, and CCLs. (A) Methylation profiles of MGMT gene obtained by Illumina arrays and expressed as relative methylation from fully unmethylated (0) to fully methylated (1). (B) Bisulfite genomic sequencing of multiple clones of the MGMT promoter in hESCs and normal primary tissues. Color code as for Fig. 1. (C) Relative expression of MGMT in hESCs and normal tissue. qPCR data are normalized with respect to GAPDH expression and presented as the percentage relative to normal lymphocytes. (0.04 MB PDF) [file pone.0003294.s002.pdf]

Figure S3

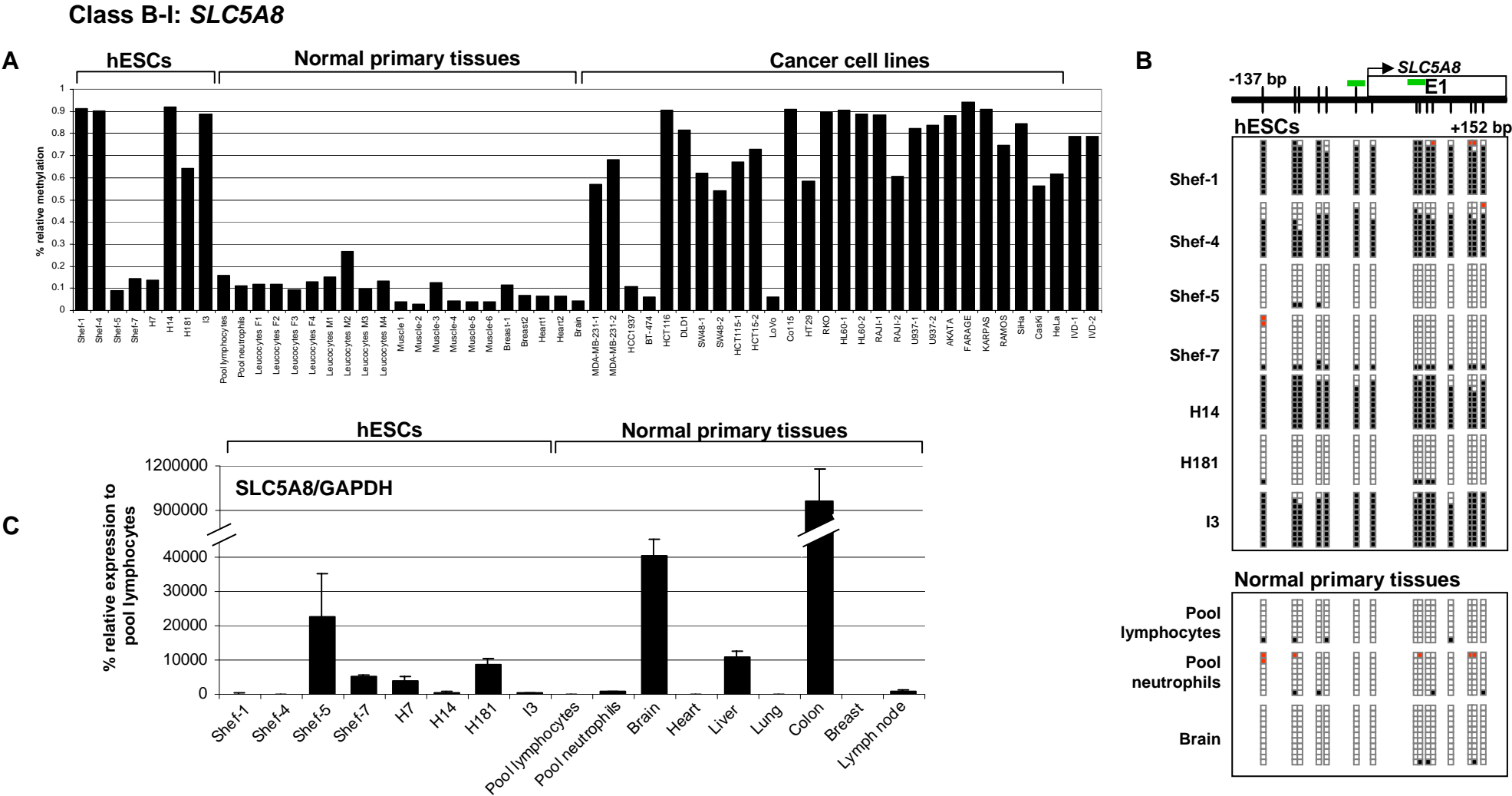

Supplement: Figure S3 — Hypermethylation of SLC5A8 in hESCs. (A) Methylation profiles of SLC5A8 gene obtained by Illumina arrays and expressed as relative methylation, from fully unmethylated (0) to fully methylated (1). (B) Bisulfite genomic sequencing of multiple clones of the SLC5A8 promoter in hESCs and normal primary tissues. Color code as for Fig. 1. (C) Relative expression of SLC5A8 in hESCs and normal tissue. qPCR data are normalized with respect to GAPDH expression and are presented as the percentage relative to normal lymphocytes. (0.03 MB PDF) [file pone.0003294.s003.pdf]

Figure S4

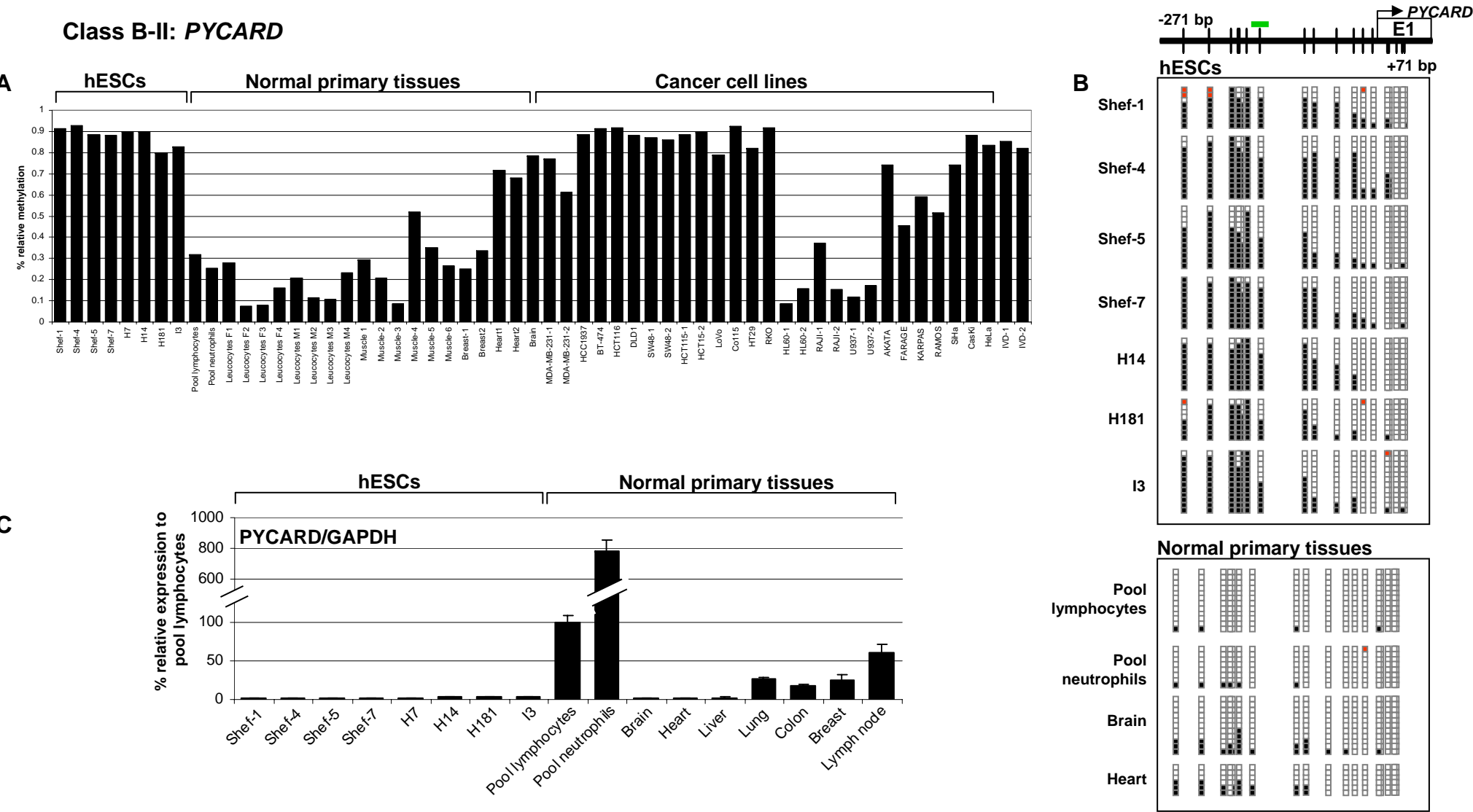

Supplement: Figure S4 — Hypermethylation of PYCARD in hESCs. (A) Methylation profiles of PYCARD gene obtained by Illumina arrays and expressed as relative methylation from fully unmethylated (0) to fully methylated (1). (B) Bisulfite genomic sequencing of multiple clones of the PYCARD promoter in hESCs and normal primary tissues. Color code as for Fig. 1. (C) Relative expression of PYCARD in hESCs and normal tissue. qPCR data are normalized with respect to GAPDH expression and are presented as the percentage relative to normal lymphocytes. (0.03 MB PDF) [file pone.0003294.s004.pdf]

Figure S5

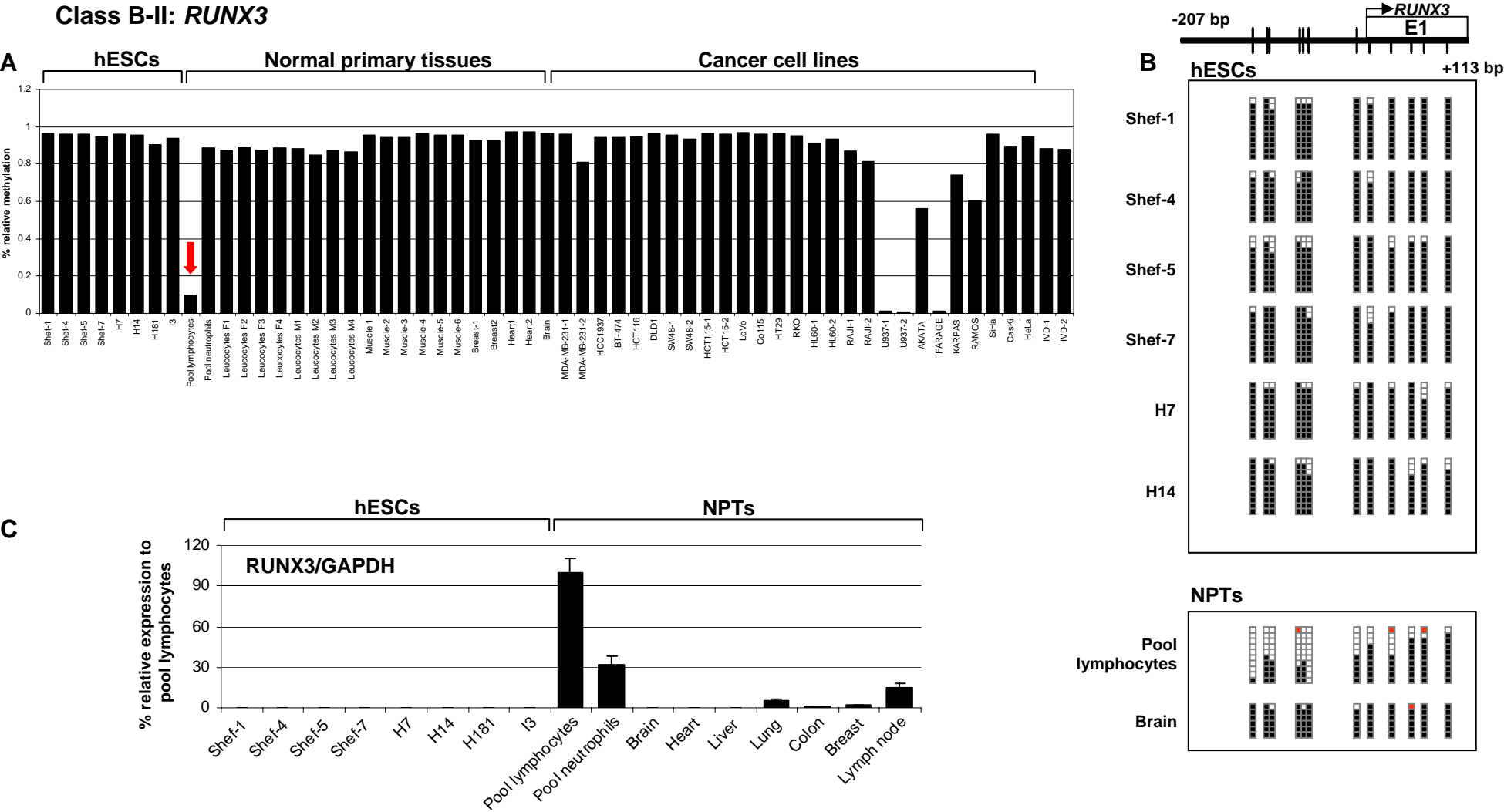

Supplement: Figure S5 — Hypermethylation of RUNX3 in hESCs. (A) Methylation profiles of RUNX3 gene obtained by Illumina arrays and expressed as relative methylation from fully unmethylated (0) to fully methylated (1). Red arrow indicates methylation levels in normal lymphocytes purified from blood. (B) Bisulfite genomic sequencing of multiple clones of the RUNX3 promoter in hESCs and normal primary tissues. Color code as for Fig. 1. (C) Relative expression of RUNX3 in hESCs and normal tissue. qPCR data are normalized with respect to GAPDH expression and are presented as the percentage relative to normal lymphocytes. (0.02 MB PDF) [file pone.0003294.s005.pdf]

Figure S6

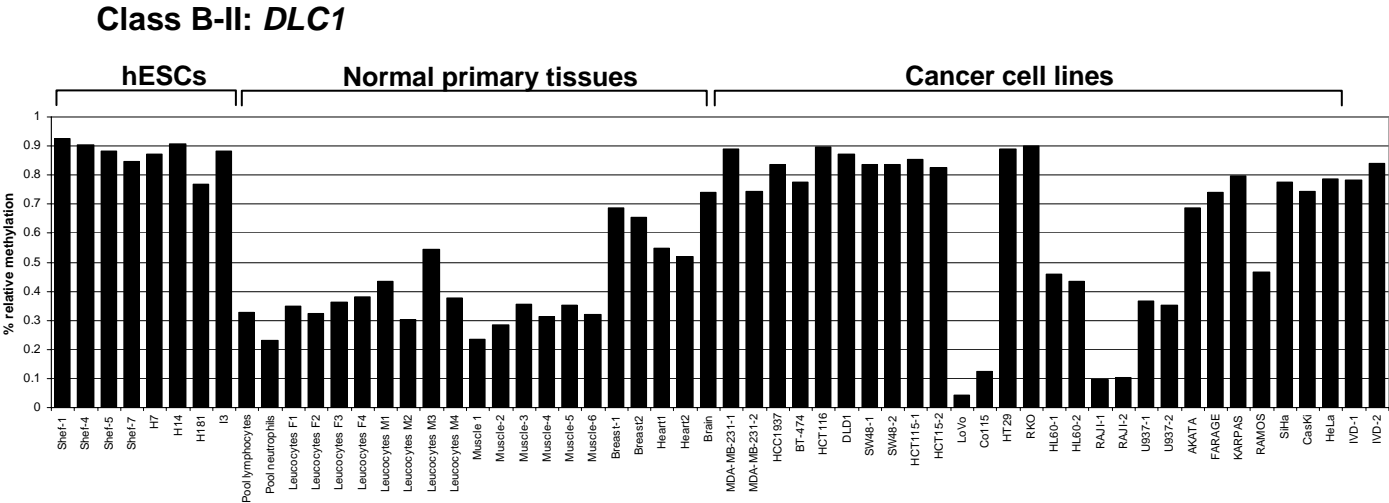

Supplement: Figure S6 — Hypermethylation of DLC1 in hESCs. Methylation profiles of DLC1 gene obtained by Illumina arrays and expressed as relative methylation, from fully unmethylated (0) to fully methylated (1). (0.02 MB PDF) [file pone.0003294.s006.pdf]

Figure S7

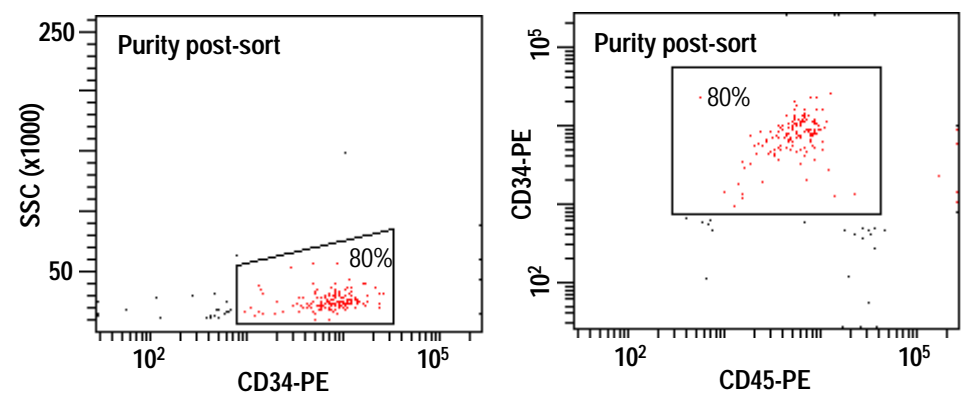

Supplement: Figure S7 — Flow cytometry analysis of the purity of CD34+ cells after purification by positive selection using anti-CD34 microbeads. Detection signals were obtained using a fluorochrome-conjugated anti-CD34 antibody (BD). Purity was 80% ± 12% (n = 2). (0.01 MB PDF) [file pone.0003294.s007.pdf]

Figure S8

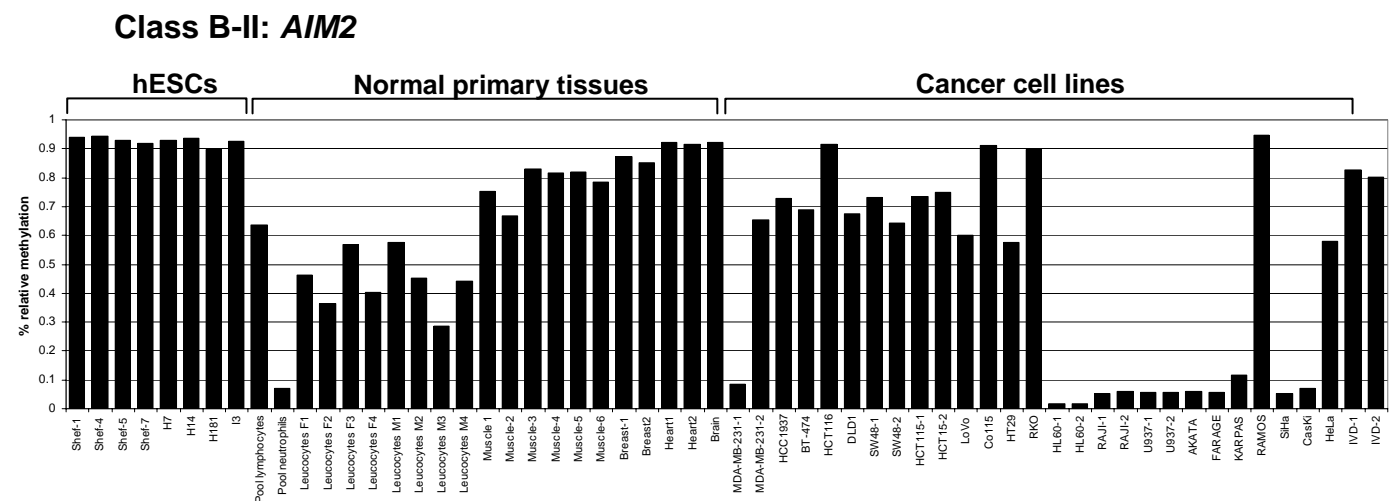

Supplement: Figure S8 — Hypermethylation of AIM2 in hESCs. Methylation profiles of AIM2 gene obtained by Illumina arrays and expressed as relative methylation, from fully unmethylated (0) to fully methylated (1). (0.02 MB PDF) [file pone.0003294.s008.pdf]
